# The challenge of conducting qualitative research to understand the factors that influence equity in medical education: A scoping review

**DOI:** 10.15694/mep.2020.000084.1

**Published:** 2020-04-30

**Authors:** John Sandars, Jeremy Brown, Chidiebere Nwolise, Mumtaz Patel, Nisha Dogra, Axel Kaehne, Jayne Garner, Simon Watmough, Michelle Maden, Vicky Duckworth

**Affiliations:** 1 Edge Hill University; 2 University of Oxford; 3 Health Education England North West; 4 University of Leicester

**Keywords:** equity, basic medical education, postgraduate medical education, qualitative research.

## Abstract

This article was migrated. The article was marked as recommended.

Introduction

There are national and international concerns about equity in basic and postgraduate medical education, especially about differential rates of access and attainment across groups of learners. Qualitative research has been increasingly used to understand the factors that influence equity but there are potential limitations to this understanding related to how the research has been conducted. The aim of the scoping review was to identify how qualitative research exploring the factors that influence equity in basic and postgraduate medical education has been conducted. The intention was to inform future research.

Methods

The electronic databases British Education Index, Campbell Library, CINAHL, Cochrane Library, EMBASE, ERIC, Google Scholar, Health Management Information Consortium (HMIC), MEDLINE, PsycINFO, Web of Science and medical education journals were searched to identify relevant published articles between 2008 and April 2019.

Results

Among 19,523 articles identified from the literature search, 72 full text articles were included in the review. Most studies had a focus on only one background characteristic and only two studies had a strengths-based focus on individuals. Recommendations for change was at the ‘policy level’ in ten studies and four studies had learner recommendations for change. No studies with a participatory approach were identified.

Conclusion

The approach to conducting previous qualitative research appears to limit greater understanding of the complexity of factors that influence equity. In response to this challenge, we recommend that future research widen the focus to consider the experiences and strengths of individual learners in addition to those identified by background characteristics. Future qualitative research is recommended to have a broad focus on both the ‘policy level’ and ‘local level’, especially from multiple perspectives. We also recommend greater collaboration of participants with researchers throughout the research process.

## Introduction

There are increasing national and international concerns about differences in both the extent and experiences of access to education and the educational outcomes of specific groups of medical students and doctors in training who are identified by their background characteristics, such as race, gender and socio-economic status ( GMC, 2017; Gardner, 2018; Woolf *et al.*, 2018). These concerns are related to two main factors: (a) an increasing global focus on equal opportunities in education (Salmi and Bassett, 2014; UNESCO, 2017), and (b) an increasing recognition that a global medical workforce that is inclusive and has diverse background characteristics is essential for effectively responding to the complex future health and social needs of both individuals and populations ( Bhutta *et al.*, 2010).

Reducing the wide global disparities in access to education and educational outcomes, both within and between countries, is a major United Nations priority and has been driven by a series of declarations from the United Nations Educational, Scientific and Cultural Organization (UNESCO). These declarations have produced a variety of national political and legal directives that are intended to influence the education systems of the country, including medical education (UNESCO, 2017). There are important philosophical and political debates about the most appropriate social justice response to these directives ( Gewirtz, 2006), but UNESCO has proposed that the focus should be on the achievement of equity (UNESCO, 2018). The key feature of equity in education is ensuring that the policies and practices of the educational system can enable all learners to be successful, irrespective of their background characteristics. This concept of equity is in contrast to equality, which has a focus on achieving equal outcomes for all learners. Equality also has important limitations when applied to education, especially if there is recognition that there are individual differences in attributes that will require considerable and sustainable use of resources to ensure that there is no disadvantage in achieving equality of access or outcomes ( Jacob and Holsinger, 2008).

### Factors influencing equity

There are a variety of factors that influence equity in medical education but a socio-ecological systems model ( Bronfenbrenner, 2009), in which the learner is embedded within the nested sub-systems of the education system, with each sub-system having an essential influence on the development and implementation of equity, can provide a useful framework to understand the factors and their inter-relationship. At the ‘policy level’, a sub-system of national political and legal directives and policies on equity influence the basic medical and postgraduate medical education systems. The ‘policy level’ interventions that influence equity include widening access policies and standards for the delivery of medical education. At the ‘local level’, the education system enacts ‘policy level’ decisions that influence the equity of medical students and doctors in training. The ‘local level’ interventions that influence equity include the various structures, procedures and processes that influence admissions, curriculum delivery and assessments. These ‘local level’ interventions directly interact with all learners, with each learner having diverse background characteristics and educational attributes, such as approaches to learning. The socio-ecological systems model highlights the complexity and importance of the interplay between the ‘policy level’ and the ‘local level’, with the ‘policy level’ influencing the ‘local level’, with all of these levels interacting with individual learners to influence the achievement of equity. At all levels, and also at the interface between levels, there are a variety of enabling and constraining factors, such as budgetary allocations and teaching staff training, that will influence how equity of education is achieved.

### Use of qualitative research for understanding equity

Understanding complex socio-ecological systems requires both quantitative and qualitative research ( Onwuegbuzie, Collins and Frels, 2013). Numerous quantitative research studies on equity in medical education have been published over the last decade and although this research can be useful to identify and monitor areas of concern, such as to highlight attainment gaps for groups of medical students with specific background characteristics, qualitative research is essential to obtain an in-depth understanding of the factors that influence equity (Strunk and Locke, 2019).

### Potential limitations of qualitative research for understanding equity

There has been increasing qualitative research on the factors that influence equity in medical education but we are increasingly aware that there may be potential limitations in how this research has been conducted, which may restrict further understanding and its application to develop and implement change at both ‘policy level’ and ‘local level’. For example, the lack of involving learners in making recommendations for change provides a limited perspective that can influence decisions about future ‘policy level’ and ‘local level’ actions for the development and improvement in equity.

In the wider field of education there has also been increasing discussion about the importance and extent to which qualitative research to understand the factors that influence equity in education accurately reflects the concerns of the learners. Participatory methodologies are widely used in education to empower individuals and groups by actively collaboratively working with researchers throughout the research process, from the initial development of focus and framing of questions to decisions about preferred methods of data collection to recommendations for change ( Atkins and Duckworth, 2019; Strunk and Locke, 2019). An important aspect of this approach to research is that the findings, which can subsequently influence educational policy and practice, accurately represent the aspirations, experiences and recommendations for change of the participants.

### Aim of the review

There has been increasing interest in ‘meta-research’, which critically evaluates how the approach to conducting research determines the findings and subsequently informs future major decisions about policy, practice and future research ( Ioannidis *et al.*, 2015; Ioannidis, 2018).

The aim of the scoping review was to identify how qualitative research exploring the factors that influence equity in basic and postgraduate medical education has been conducted. The objectives were to identify the focus of the research, the sub-system level of the main recommendations for action, and the active participation of medical students and junior doctors in training in the research process.

## Methods

A scoping review methodology was selected since it provides a “map” of the breadth of literature within a particular field, with the identification of the extent and range of research available on a given topic. Our review followed ‘best practice’ in performing scoping reviews and met the criteria of the PRISMA-Scoping Review (PRISMA-ScR) guidelines (Tricco *et al*., 2018). We followed the five-stage process for scoping reviews outlined by Arksey and O’Malley (2005): [1] identifying the research question, [2] identifying potentially relevant articles, [3] selecting articles, [4] charting data, [5] collating, summarising and reporting the results. The review also adhered to the recommendations of Levac, Colquhoun and O’Brien (2010) for ensuring the quality of scoping reviews, with a team of multi-disciplinary researchers, a transparent and replicable process with regular team meetings, review of full articles for inclusion, and a descriptive summary of the evidence.

This study was registered with the Faculty of Health, Social Care & Medicine at Edge Hill University. Further ethical scrutiny was not applicable as this was a scoping review and did not involve primary data collection.

### Identifying relevant articles

We began by establishing a research team of reviewers with expertise in basic medical and postgraduate medical education (JB, JS, SW), equity and social justice in basic medical and postgraduate medical education (ND, JG, MP), equity and social justice in general education (VD) and systematic reviews (AK, CN, MM). We held an initial meeting with all reviewers during which we determined the research questions and drafted the scoping review protocol.

The scoping review search strategy was developed by the research team that included an experienced information specialist (MM). Search terms were identified via key relevant studies and a published health inequalities search filter (Welch *et al.*, 2016). The search strategy was initially developed in MEDLINE using a combination of controlled vocabulary and free-text keywords. A small set of key relevant articles was identified, and the initial MEDLINE search strategy was tested to ensure it captured all relevant studies. Underpinned by the inclusion criteria, the search was structured using three main concepts: [1] terms relating to equity, [2] terms relating to medical education and [3] terms relating to qualitative research. The MEDLINE search was then translated into other databases; a comprehensive search strategy was developed to ensure maximum sensitivity.

Only published literature was searched from January 2008 onwards to April 2019 since it was our intention to focus on recently conducted research studies so that we could obtain a ‘snapshot’ of previous research practice that would be highly relevant to inform recommendations for conducting future research studies. The following databases were searched in May 2019: British Education Index, Campbell Library, CINAHL, Cochrane Library, EMBASE, ERIC, Google Scholar, Health Management Information Consortium (HMIC), MEDLINE, PsycINFO, Web of Science. In addition to a citation search of included studies, targeted searches were undertaken in Google Scholar, on key authors in the field and in four key journals (Academic Medicine, Medical Education, Medical Teacher, Advances in Health Sciences Education). Citations from the databases and journals were imported into Covidence (a Cochrane systematic review technology platform: Melbourne,VIC: Australia) and this platform was also used for screening and selection of studies.

### Selecting studies for review

A two-stage screening process was used to determine the relevance of studies. For the first stage of screening, two reviewers (JB and CN) independently reviewed the titles and abstracts, any disagreements were resolved through discussion and consensus. Articles were excluded if there was an agreement that they met one or more of the following exclusion criteria: quantitative studies, descriptive articles, letters to the editor, commentaries, book chapters, grey literature or non-medical student population. For a study to be included in the scoping review, it must be: a peer reviewed qualitative or mixed methods study in English language with a focus on an aspect of equity in basic medical and postgraduate medical education. For the second stage of screening, the full articles for all studies for potential inclusion were independently reviewed by pairs of reviewers (JB, ND, JG, MP, JS, SW), with any disagreements resolved through discussion and consensus.

### Charting the data

A data extraction sheet was jointly developed by three reviewers (JB, CN and JS) to determine which data to extract. One reviewer (CN) initially charted five studies to ensure that the extraction sheet was fit for purpose and following a discussion with two reviewers (JB and JS), the sheet was amended. Data were initially charted by one reviewer (CN) and each study was verified for accuracy by seven other reviewers working in pairs [JB, ND, JG, MP, JS, SW], with any disagreements resolved through discussion and consensus. Descriptive data were collected for each study to identify the focus, the sub-system level of the main recommendations for action, and the active participation of medical students and junior doctors in training in the research process.

### Collating, summarizing and reporting findings

We classified studies into the phases of basic medical education or postgraduate medical education, and within each category as entry (such as widening access to medical school or selection for postgraduate medical training) or curriculum (which we considered as participation in basic medical or postgraduate medical education, such as the interaction with teachers and others in the academic and clinical environment, but also assessment and support). Data were initially charted by one reviewer (CN) and each study was verified for accuracy by seven other reviewers working in pairs (JB, ND, JG, MP, JS, SW), with any disagreements resolved through discussion and consensus.

## Results

A total of 19,523 articles were identified from the literature search and after removing duplicates, the titles and abstracts of 12,457 articles were screened for eligibility. After excluding 12,364 articles that did not meet the eligibility criteria, 93 full text articles were selected for detailed review, of which 72 met the eligibility criteria.

**Figure 1.**
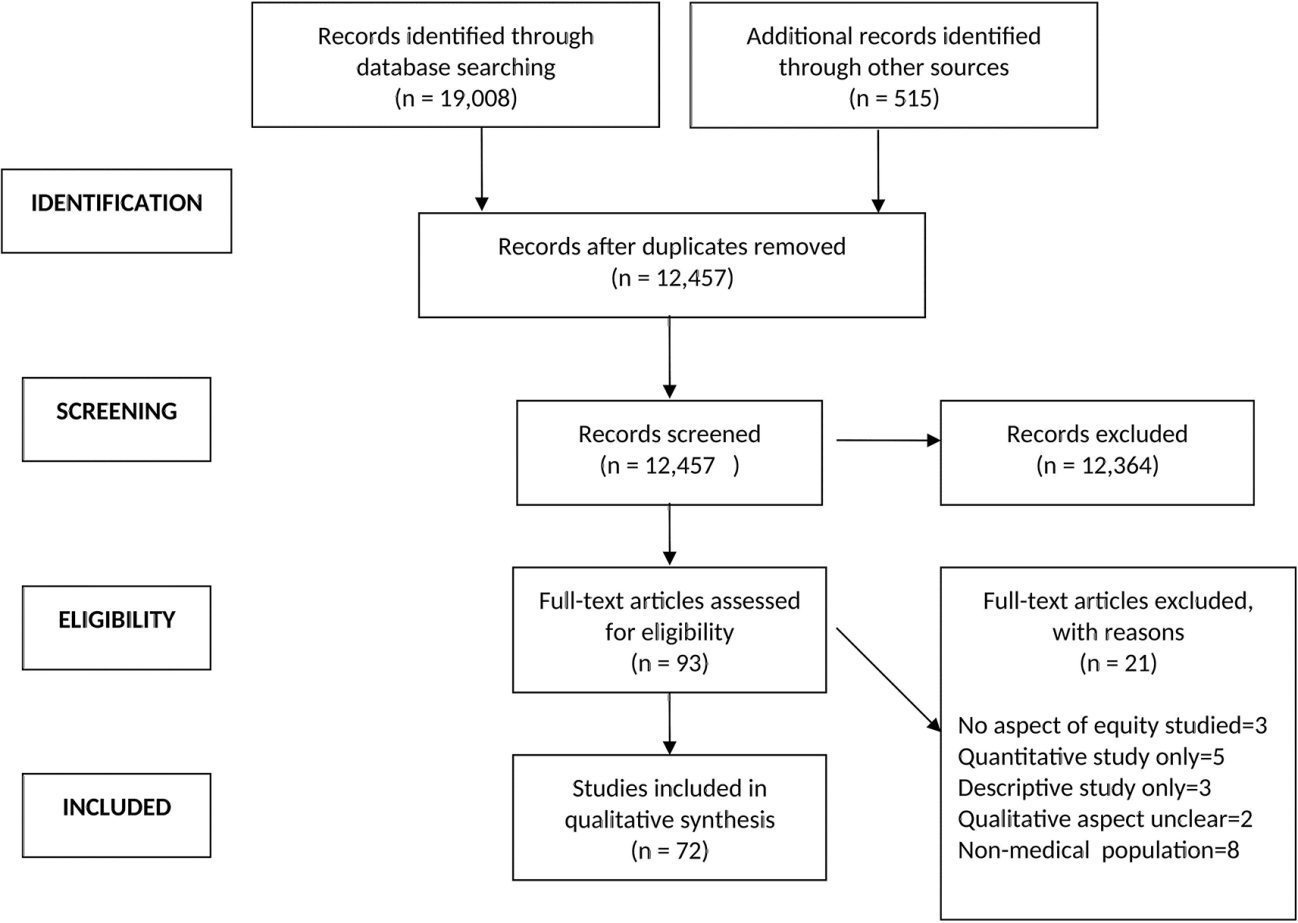
PRISMA flow diagram

Please see Figure 1: Preferred reporting items for systematic reviews and meta-analysis (PRISMA) flow diagram ( Moher *et al.*, 2009).

The list of the included studies in the review is provided in the Appendix.

### Focus of studies

(a) Background characteristics

Most studies had a focus on a single background characteristic of the medical students and junior doctors in training [n=62], with the remaining studies having a focus on a combination of background characteristics.

For basic medical education entry, the main focus was socioeconomic status [n=14], with ‘under-represented minority’ students as the next most frequent group [n=11]. The main areas of focus for basic medical education curriculum were ‘under-represented minority’ students [n=4], race or ethnicity [n=7] and female students [n=4]. Four studies compared the experience of female with male students, and two studies had a focus on black male students.

In postgraduate medical education, the only study with a focus on entry was on international medical graduates. For postgraduate medical education curriculum, the main areas of focus were international medical graduates [n=7] and comparison of the experiences and achievements of female with males [n=4].

Please see Table 1: Focus - Background Single Characteristic and Table 2: Focus - Background Multiple Characteristics.

**Table 1.** Focus - Background Single Characteristic

Background Characteristic	Basic Medical EducationENTRY	Basic Medical EducationCURRICULUM	Postgraduate Medical EducationENTRY	Postgraduate Medical Education CURRICULUM
Socio-economic status (SES)	N=142, 11, 16, 17, 23, 35, 36, 37, 42, 43, 53, 54, 70, 71			
First in Family	N=256, 57	N=39, 13, 57		
Race or ethnicity		N=73, 14, 26, 27, 33, 68, 72		N=240, 61
Under Represented Minority (URM)	N=718, 22, 24, 44, 60, 65, 66	N=47, 8, 20, 65		
International Medical Graduates (IMG)			N=167	N=73, 15, 21, 49, 55, 67, 69
Female Only		N=225, 32		
Female compared with male		N=429, 30, 38, 64		N=428, 38, 39, 58
Black Male	N=162	N=21, 62		
Sexual Orientation		N=134		
Dyslexia		N=250, 52		N=241, 51
Disability		N=259, 63		
Harassment		N=112		
‘Diversity’ climate	N=445, 46, 47, 48	N=219		

* Two studies include both Basic Medical Education Entry and Curriculum, one study includes both Postgraduate Medical Education Entry and Curriculum and one study includes both Basic Medical Education Curriculum and Postgraduate Medical Education Curriculum.

* The numbers refer to the study in the list of included studies presented in Appendix.

**Table 2.** Focus - Background Multiple Characteristics

Background Characteristic	Basic Medical Education ENTRY	Basic Medical Education CURRICULUM	Postgraduate Medical Education ENTRY	Postgraduate Medical Education CURRICULUM
Mixed gender /race /sexuality		N=44, 5, 6, 19		N=110
Mixed socio-economic status (SES)/ethnicity	N=131			

*The numbers refer to the study in the list of included studies presented in Appendix.

(b) Sub-system level

Most studies had a focus on the ‘local level’ sub-system to identify the perceptions and experiences of medical students and junior doctors in training about entry and the curriculum [n=65]. These studies mainly identified factors that constrained individual potential but there were two studies that had a specific focus on factors that enabled individual student success and one on the cultural assets that students bring to medical education.

Seven studies on basic medical education entry had a ‘policy level’ focus, including the tension between the political and legal directives and policies and their enactment by the medical school.

Please see Table 3: Focus - Sub-system level

**Table 3.** Focus - Sub-system level

Sub-system level	Studies
Policy-level	N=72 16, 42, 45, 46, 47, 57
Local-level	Focus on perceptions and/or experiences of the curriculum N=651,3, 4 5 ,6 ,7 ,8, 9 ,10, 11, 12, 13, 14, 15, 17, 18, 19, 20, 21, 22, 23, 24, 25, 26, 27, 28, 29, 30, 31, 32, 33, 34, 35, 36, 37, 38, 39, 40, 41, 43, 44, 48, 49 50, 51, 52, 53, 54 ,55, 56 ,58, 59, 60, 61, 62, 63, 64, 65, 66, 67, 68, 69, 70, 71, 72 Focus with a specific focus on positive aspects of the learning environment N=3 Success stories 36, 62 Cultural assets 72

* The numbers refer to the study in the list of included studies presented in Appendix.

### Recommendations for change

All studies had researcher proposed recommendations for change at the ‘local level", and recommended changes in the ‘policy level’ were discussed in twenty one studies. The areas discussed included the tensions of increasing diversity and the maintenance of academic standards, the complex contribution of wider societal factors that contribute to differences in secondary educational opportunities and achievement related to medical students characterised by specific racial and socio-economic characteristics, and funding priorities and allocations for medical schools.

Please see Table 4: Recommendations for change.

**Table 4.** Recommendations for change

Recommendation	Studies
Researcher based recommendations for change - policy level	N=616, 36, 38, 45, 46, 47, 56, 57, 61, 64, 71
Researcher based recommendations for change - local level	N= 2010, 12, 19, 20, 21, 29, 30, 33, 34, 38, 40, 41, 50, 52, 59, 63, 65, 69, 72
Participant based recommendations for change - policy level	N=0
Participant based recommendations for change - local level	N= 412, 20, 21, 50

* The numbers refer to the study in the list of included studies presented in Appendix

Active participation of learners in the research process

No studies were identified in which medical students and junior doctors in training were discussed as being involved in the choice of focus or methodology except for one auto-ethnographic study.

Participant proposed recommendations for change at the ‘local level’ were discussed in four studies with a focus on the learning environment. No studies were identified in which medical students and junior doctors in training made recommendations for change at the ‘policy level’ sub-system.

***Supplementary data*** ***available online:*** Supplementary File 1: Extracted data from the included studies

• Focus: (a) country (b) phase of medical education
• Qualitative research methods: Data collection

## Discussion

To our knowledge, this is the first overview of how qualitative research exploring the factors that influence equity in basic and postgraduate medical education has been conducted. Our findings have highlighted that the approach to conducting previous qualitative research appears to limit understanding of the complexity of the factors that influence equity, with a potential impact on appropriate decision-making about future policy and practice to improve equity in medical education.

All identified studies only had a focus of interest on a group of medical students and junior doctors in training that had been identified by their background characteristics, and most studies had only a focus on a single background characteristic. This focus is likely to be highly influenced by national and /or local interests in relation to policy directives and also the specific interests of the researcher. For example, the studies of ‘first in family’ in medical students appear to have been initiated in response to national policy in Australia.

Defining a study by a focus on a group with a specific background characteristic, such as ‘first in family’, has several advantages. The advantages include increasing awareness to educators and policy makers about inequity in a specific group and the practical aspect of ease of selection of participants to a study. However, there are several potentially important limitations to understanding equity by having a focus on a group with a specific background characteristic. These limitations include:

• the classification of a group with a background characteristic, such as socio-economic status, may be contested, with a lack of consistent definition being used by both researchers and policy makers who are interested in the findings( Burlew *et al.*, 2019).
• the groups with a background characteristic may compete to be considered as the most important for attention by both researchers and policy makers, with less attention given to other groups ( Dhamoon, 2011).
• within each group with a background characteristic there will be individuals who are a minority, with the consequence that this minority subgroup may ultimately become “invisible” to the attention of both researchers and policy makers ( Purdie-Vaughns and Eibach, 2008). Feminist and anti-racist researchers initially highlighted this important consideration, which they called intersectionality, in their quest to understand issues related to fairness, noting that sexual oppression was also usually experienced simultaneously with disadvantage that was interrelated to race and socio-economic background, but the importance of these other factors were not further discussed ( Collins and Bilge, 2016).
• there is a wider moral and political philosophical aspect of equity that emphasises that each individual will have their own preferences and choices about their education ( Boyadjieva and Ilieva-Trichkova, 2018). A focus on a group with a specific background characteristic is likely to have the results presented and discussed as though there is homogeneity across the group, although there could be a variation across the individuals in the group.

Consideration of the socio-ecological system of medical education increases understanding of how the interplay of the various factors, both within the ‘policy level’ and the ‘local level’ sub-systems and between these sub-systems, enable and constrain equity ( Onwuegbuzie, Collins and Frels, 2013). A few recent studies that were identified in the review have taken this approach in relation to medical school entry, including the enactment of widening access policy though web sites, documentary analysis and interviews with key admissions staff, and also the influence of career advisors on medical student aspirations. However, most of the identified studies had a focus on the ‘local level’ sub-system but not the important influence of the external ‘policy level’, and its enactment, on medical education at the ‘local level’ to improve the learning environment.

A lack of active collaboration and participation with medical students and junior doctors in training was noted throughout the qualitative research process described in the studies, from initial setting of the focus through to making recommendations for change. There are additional considerations about the insider/outsider relationship of the researcher with the participant ( Ioannidis, 2015; Strunkand Locke, 2019). This relationship is complex since an outsider researcher relationship, in which the researcher has different characteristics to the participant, may limit free disclosure of information but an insider, with similar characteristics, can facilitate disclosure, although they may have beliefs that can influence the analysis and interpretation of findings to support their beliefs. Researchers in education have highlighted the importance of reflexivity, with a critical and heightened awareness of their relationship with their participants ( Atkins and Duckworth, 2019; Strunk and Locke, 2019).

### Limitations

There are several potential limitations to this scoping review. Research was restricted to only published qualitative studies that were indexed in major databases and there may be additional relevant un-published qualitative studies, such as PhD dissertations and internal reports. Studies may have also been missed because of the extensively different terms used to describe equity, and both selection and analysis of the included studies required individual judgments by the reviewers. We minimised this potential limitation by adopting a systematic and expert informed process to develop our search strategy, and we also attempted to reduce variation in reviewer judgments by the use of pairs of reviewers who discussed any differences to achieve consensus.

Similar to all scoping reviews, an exhaustive search of all potential sources of studies was not performed but our approach was rigorous and we identified important themes that are of interest to guide further research.

### Recommendations for future research

Our findings have informed our recommendations for conducting future qualitative research to understand the factors that influence equity in medical education, with the intention that future research can improve equity for medical students and junior doctors identified by background characteristics but also for all learners.

We recommend that the focus of research is widened to achieve greater understanding of the diversity of the individual experience of all medical students and junior doctors in training, including those individuals with background characteristics. This recommendation resonates with other education researchers interested in understanding and achieving equity ( Atkins and Duckworth, 2019; Strunk and Locke, 2019). An important theoretical perspective and methodology that has increasingly informed research to understand the factors that influence equity in higher education has been the capability approach, with a focus on the factors (called ‘conversion factors’ in the capability approach) that enable and constrain the choice and achievement of the preferences that an individual has about their education (Wilson-Strydom, 2015; Harrison *et al.*, 2018; Clark, Biggeri and Frediani, 2019). This approach can also reveal the intersectionality tensions between the conversion factors if an individual has two or more background characteristics ( Núñez, 2014).

We recommended future research that has a focus on the important interplay of the various factors within, and between, the ‘policy level’ and ‘local level’ sub-systems. The achievement of equity in education can only occur if there is an integrated and continuous approach within the education system that has a focus on all learners and considers the wide variety of factors, at both ‘policy level’ and ‘local level’, that enable and constrain equity. This will require future research to obtain the views and experiences of the multiple perspectives of the wide variety of stakeholders, including programme and course managers at the ‘policy level’ to teachers and learners at the ‘local level’. Further insights can also be obtained by considering intersectionality tensions if an individual has two or more background characteristics especially between the tensions at ‘policy level’ and ‘local level’ ( Núñez, 2014).

An important consideration for further qualitative research on the factors that influence equity in medical education is the recognition that some individuals can gain advantage from the interplay of the various factors within a socio-ecological system ( Collins and Bilge, 2016). We recommend that future research includes strengths-based methodologies, such as through appreciative inquiry, which can offer opportunities to not only identify individual strengths but also empower individuals to make changes that overcome the constraints within their socio-ecological system. In addition, we recommend that future research also has an increased focus on the positive aspects of the learning environment that enable equity.

We also recommend there is an increasing emphasis on the active involvement of participants throughout the entire research process to ensure that their essential perspective can inform future decisions on policy and practice ( Atkins and Duckworth, 2019; Strunk and Locke, 2019). This will require medical education researchers to increase their use of participatory research methodologies and methods. Two important aspects of participatory research in education are the increased use of data collection methods that are chosen by participants, such as social media or photographs, and widening the variety of participants who represent the socio-ecological system, including learners, teachers and national policy makers. These approaches can also empower individuals to make the essential changes to improve equity.

## Conclusions

Our review has highlighted that the approach to conducting previous qualitative research appears to limit greater understanding of the complexity of the different factors that influence equity in medical education. We recommend that researchers widen the focus to consider the experiences of individual learners in addition to those identified by their background characteristics, especially when there is a focus on only a single characteristic. We also recommend greater focus on the strengths of the individual learner, a broader focus on both the ‘policy level’ and ‘local level’ sub-system, and increased involvement of medical students and junior doctors in training by active participation throughout the entire research process. Further research that is informed by these recommendations, which were derived from the findings of our review, has the potential to improve equity in basic and postgraduate medical education by responding to the challenge of promoting greater understanding of the complexity of the factors that enable and constrain equity. The ultimate intention is to increase the capacity of all medical educators to make appropriate decisions about policy and practice.

## Take Home Messages

• Qualitative research has been increasingly used to understand the factors that influence equity in medical education.
• The approach to conducting previous qualitative research appears to limit understanding of the complexity of factors that influence equity.
• Recommendations for future qualitative research include widening the focus to consider the strengths of individual learners and having a broad focus on both the ‘policy level’ and ‘local level’, especially from multiple perspectives.
• Greater collaboration of participants with researchers throughout the qualitative research process is recommended.
• Future qualitative research that is informed by the recommendations has the potential to improve equity by creating greater understanding to inform future policy and practice.

## Notes On Contributors

John Sandars is Professor of Medical Education in the Health Research Institute, Faculty of Health, Social Care & Medicine, Edge Hill University, Ormskirk, UK. ORCID iD: https://orcid.org/0000-0003-3930-387X

Jeremy Brown is Professor of Clinical Education in the Health Research Institute, Faculty of Health, Social Care & Medicine, Edge Hill University, Ormskirk, UK. ORCID iD: https://orcid.org/0000-0002-0653-4615

Chidiebere Nwolise was a Research Assistant at Edge Hill University, Ormskirk, UK and is now a Research Officer at the Health Services Research Unit, Nuffield Department of Population Health, University of Oxford, Oxford, UK. ORCID iD: https://orcid.org/0000-0002-5510-1103

Mumtaz Patel is Associate Postgraduate Dean, Health Education England North West, Manchester, UK and Clinical Lead for Quality Management for the Joint Royal Colleges of Physicians Training Board, UK. ORCID iD: https://orcid.org/0000-0001-7016-819X

Nisha Dogra is Emeritus Professor of Psychiatry Education at the University of Leicester, Greenwood Institute for Child Health, Leicester, UK and is Associate Dean for Diversity, Equality and Inclusion, Royal College of Psychiatrists, UK. ORCID iD: https://orcid.org/0000-0002-0245-6816

Axel Kaehne is Reader in Health Services Research at the Health Research Institute, Faculty of Health, Social Care & Medicine, Edge Hill University, Ormskirk, UK and Director of the Evaluation and Policy Analysis Research Unit, Edge Hill University, Ormskirk, UK. ORCID iD: https://orcid.org/0000-0002-7978-2214

Jayne Garner is Senior Lecturer in Medical Education and Director of Widening Participation at the Medical School, Health Research Institute, Faculty of Health, Social Care & Medicine, Edge Hill University, Ormskirk, UK. ORCID iD: https://orcid.org/0000-0001-7454-5140

Simon Watmough is Senior Lecturer in Medical Education and Associate Head of Undergraduate Medicine & MBChB Programme Leader at the, Medical School, Health Research Institute. Faculty of Health, Social Care & Medicine, Edge Hill University, Ormskirk, UK. ORCID iD: https://orcid.org/0000-0002-2961-8555

Michelle Maden is an Information Specialist at the Faculty of Health, Social Care & Medicine, Edge Hill University, Ormskirk, UK. ORCID iD: https://orcid.org/0000-0003-4419-6343

Vicky Duckworth is Professor in Education at the Faculty of Education, Edge Hill University, Ormskirk, UK and co-convenor of the Social Justice Special Interest Group of the British Education Research Association. ORCID iD: https://orcid.org/0000-0002-6769-425X

## Appendices

List of included studies

1. Acheampong, C., Davis, C., Holder, D. and Averett ,P. *et al.*(2019) ‘An Exploratory Study of Stress Coping and Resiliency of Black Men at One Medical School: A Critical Race Theory Perspective’, *Journal of Racial and Ethnic Health Disparities*,6(1), pp.214-219. https://doi.org/10.1007/s40615-018-0516-8.
2. Alexander, K., Fahey Palma, T., Nicholson, S. and Cleland, J. (2017) ‘’Why not you?’ Discourses of widening access on UK medical school websites’, *Medical Education*, 51(6), pp.598-611. https://doi.org/10.1111/medu.13264.
3. Anderson, C., Lee, K., Wakeling, J. and Bowie, P. (2017) ‘An enhanced induction programme for general practice specialty training: a qualitative study of trainee perceptions and experience’ *Education for Primary Care*, 28(2), pp.102-110. https://doi.org/10.1080/14739879.2017.1278621.
4. Babaria, P., Abedin, S. and Nunez-Smith, M. (2009) ‘The effect of gender on the clinical clerkship experiences of female medical students: Results from a qualitative study’, *Academic Medicine*, 84(7), pp.859- 866. https://doi.org/10.1097/ACM.0b013e3181a8130c.
5. Babaria, P., Abedin S. and Nunez-Smith, M. (2011) ‘Gender and the pre‐clinical experiences of female medical students: a taxonomy’, *Medical Education,* 45(3), pp.249-260. https://doi.org/10.1111/j.1365-2923.2010.03856.x.
6. Babaria, P., Abedin, S., Berg, D. and Nunez-Smith, M. (2012) ‘"I’m too used to it": A longitudinal qualitative study of third year female medical students’ experiences of gendered encounters in medical education’, *Social Science and Medicine*, 74(7), pp. 1013-1020. https://doi.org/10.1016/j.socscimed.2011.11.043.
7. Barr, D.A., Gonzalez, M.E. and Wanat, S.F.(2008) ‘The leaky pipeline: Factors associated with early decline in interest in premedical studies among underrepresented minority undergraduate students’, *Academic Medicine,* 83(5), pp.503- 511. https://doi.org/10.1097/ACM.0b013e31816bda16.
8. Barr, D.A., Matsui, J., Wanat, S.F. and Gonzalez, M.E.(2010) ‘Chemistry courses as the turning point for premedical students’, *Advances in Health Sciences Education Theory and Practice,* 15(1), pp. 45-54. https://doi.org/10.1007/s10459-009-9165-3.
9. Bassett, A.M., Brosnan, C., Southgate, E. and Lempp, H.(2018) ‘Transitional journeys into, and through medical education for First-in-Family (FiF) students: a qualitative interview study’, *BMC Medical Education*,18,102. https://doi.org/10.1186/s12909-018-1217-z.
10. Bezuidenhout, J., Cilliers, F., Van Heusden, M. and Wasserman, E. *et al.* (2011) ‘Alienation and engagement in postgraduate training at a South African medical school’, *Medical Teacher,* 33(3), pp.e145-153. https://doi.org/3109/0142159X.2011.543198.
11. Biggs, L., Grafton-Clarke, C. and Garner, J. (2019) ‘Perception of medicine in under-represented students’, *Education for Primary Care*, 30(1), pp.49-51. https://doi.org/10.1080/14739879.2018.1540946 .
12. Broad, J., Matheson, M., Verrall, F. and Taylor, A.K. *et al.* (2018) ‘Discrimination, harassment and non‐reporting in UK medical education’, *Medical Education*, 52(4), pp.414- 426. https://doi.org/10.1111/medu.13529.
13. Brosnan, C., Southgate, E., Outram, S. and Lempp, H. *et al.* (2016) ‘Experiences of medical students who are first in family to attend university’, *Medical Education*, 50(8), pp.842-851. https://doi.org/10.1111/medu.12995.
14. Chandauka, R.E., Russell, J.M., Sandars, J. and Vivekananda-Schmidt, P. (2015) ‘Differing perceptions among ethnic minority and Caucasian medical students which may affect their relative academic performance’, *Education for Primary Care* *, *26(1), pp.11-15. https://doi.org/10.1080/14739879.2015.11494301.
15. Chen, P.G., Curry, L.A., Bernheim, S.M. and Berg, D. *et al.* (2011) ‘Professional challenges of non-US-born international medical graduates and recommendations for support during residency training’, *Academic Medicine*, 86(11), pp 1383-1388. https://doi.org/10.1097/ACM.0b013e31823035e1.
16. Cleland, J.A., Nicholson, S., Kelly, N. and Moffat, M. (2015) ‘Taking context seriously: explaining widening access policy enactments in UK medical schools’, *Medical Education*, 49(1), pp. 25-35. https://doi.org/10.1111/medu.12502.
17. Cleland, J and Palma, T.F. (2018) ‘"Aspirations of people who come from state education are different": how language reflects social exclusion in medical education’, *Advances in Health Sciences Education Theory and Practice*, 23(3), pp. 513-531. https://doi.org/10.1007/s10459-018-9809-2.
18. Derck, J., Zahn, K., Finks, J.F. and Mand, S. *et al.* (2016) ‘Doctors of tomorrow: an innovative curriculum connecting underrepresented minority high school students to medical school’, *Education for Health (Abingdon),* 29(3), pp.259-265. http://www.educationforhealth.net/text.asp?2016/29/3/259/204219 (Accessed: 30 January 2019).
19. Dhaliwal, J.S., Crane, L.A., Valley, M.A. and Lowenstein, S.R. (2013) ‘Student perspectives on the diversity climate at a US medical school: the need for a broader definition of diversity’, *BMC Research Notes*, 6,154. https://doi.org/10.1186/1756-0500-6-154.
20. Dickins,K., Levinson, D., Smith, S.G. and Humphrey, H.J.(2013) ‘The minority student voice at one medical school: lessons for all?’, *Academic Medicine*, 88(1), pp.73-79. https://doi.org/10.1097/ACM.0b013e3182769513.
21. Dorgan, K.A., Lang, F., Floyd, M. and Kemp, E. (2009) ‘International medical graduate-patient communication: A qualitative analysis of perceived barriers’, *Academic Medicine*, 84(11),pp.1567-1575. https://doi.org/10.1097/ACM.0b013e3181baf5b1.
22. Freeman,B.K., Landry,A., Trevino,R.and Grande,D. *et al*.(2016)’Understanding the leaky pipeline: perceived barriers to pursuing a career in medicine or dentistry among underrepresented-in-medicine undergraduate students’ *, Academic Medicine*, 91(7), pp.987-993. https://doi.org/10.1097/ACM.0000000000001020.
23. Grafton-Clarke, C., Biggs, L. and Garner, J. (2018) ‘Why students from under-represented backgrounds do not apply to medical school’, *Widening Participation and Lifelong Learning*, 20(1), pp.187-198. https://doi.org/10.5456/WPLL.20.1.187.
24. Hadinger, M.A.(2017) ‘Underrepresented minorities in medical school admissions: a qualitative study’, *Teaching and Learning in Medicine,* 29(1), pp.31-41. https://doi.org/10.1080/10401334.2016.1220861.
25. Hill, E. and Vaughan, S. (2013) ‘The only girl in the room: how paradigmatic trajectories deter female students from surgical careers’, *Medical Education,* 47(6), pp.547-556. https://doi.org/10.1111/medu.12134.
26. Huhn, D., Eckart, W., Karimian-Jazi, K. and Amr, A. *et al.* (2014) ‘Voluntary peer-led exam preparation course for international first year students: Tutees’ perceptions’, *BMC Medical Education*, 15, 106. https://doi.org/10.1186/s12909-015-0391-5.
27. Huhn, D., Huber, J., Ippen, F.M. and Eckart, W. *et al.* (2016) ‘International medical students’ expectations and worries at the beginning of their medical education: a qualitative focus group study’, *BMC Medical Education*,16,33. https://doi.org/10.1186/s12909-016-0549-9.
28. Kobayasi, R., Tempski, P.Z., Arantes-Costa, F.M. and Martins, M.A. (2018) ‘Gender differences in the perception of quality of life during internal medicine training: a qualitative and quantitative analysis’, *BMC Medical Education,* 18,281. https://doi.org/10.1186/s12909-018-1378-9.
29. Kristoffersson, E., Andersson, J., Bengs, C. and Hamberg, K. (2016) ‘Experiences of the gender climate in clinical training-a focus group study among Swedish medical students’, *BMC Medical Education*,16,283. https://doi.org/10.1186/s12909-016-0803-1.
30. Kristoffersson, E., Diderichsen, S., Verdonk, P. and Lagro-Janssen, T. *et al.* (2018) ‘To select or be selected-gendered experiences in clinical training affect medical students’ specialty preferences. *BMC Medical Education.*2018;18(1):268. https://doi.org/10.1186/s12909-018-1361-5.
31. Leduc, J.M., Rioux, R., Gagnon, R. and Bourdy, C. *et al.* (2017) ‘Impact of sociodemographic characteristics of applicants in multiple mini-interviews *’, Medical Teacher*, 39(3), pp.285-294. https://doi.org/10.1080/0142159X.2017.1270431.
32. Levine, R.B., Mechaber, H.F., Reddy, S.T. and Cayea, D. *et al.* (2013) ‘"A Good Career Choice for Women": Female Medical Students’ Mentoring Experiences A Multi-Institutional Qualitative Study’, *Academic Medicine,* 88(4), pp.527-534. https://doi.org/10.1097/ACM.0b013e31828578bb.
33. Leyerzapf, H. and Abma, T. (2017) ‘Cultural minority students’ experiences with intercultural competency in medical education’, *Medical Education*, 51(5), pp.521-30. https://doi.org/10.1111/medu.13302.
34 Mansh, M., White, W., Gee-Tong, L. and Lunn, M.R. *et al.* (2015) ‘Sexual and gender minority identity disclosure during undergraduate medical education: “In the closet” in medical school’, *Academic Medicine,* 90(5), pp.634-644. https://doi.org/10.1097/ACM.0000000000000657.
35 Martin, A.J., Beska, B.J., Wood, G. and Wyatt, N. *et al.* (2018) ‘Widening interest, widening participation: factors influencing school students’ aspirations to study medicine’. *BMC Medical Education*,18:117. https://doi.org/10.1186/s12909-018-1221-3.
36 Mathers, J and Parry, J. (2006) ‘Why are there so few working‐class applicants to medical schools? Learning from the success stories’, *Medical Education*, 43(3), pp.219-228. https://doi.org/10.1111/j.1365-2923.2008.03274.x.
37 Mathers, J and Parry, J. (2010) ‘Older mature students’ experiences of applying to study medicine in England: an interview study’, *Medical Education*, 44(11), pp.1084-1094. https://doi.org/10.1111/j.1365-2923.2010.03731.x.
38 Miller, K. and Clark, D. (2008) ‘"Knife before wife": An exploratory study of gender and the UK medical profession’, *Journal of Health Organization and Management,* 22(3), pp.238-253. https://doi.org/10.1108/14777260810883521.
39 Morris, L., Cronk, N.J. and Washington, K.T.(2016) ‘Parenting during residency: providing support for Dr Mom and Dr Dad’, *Family Medicine,* 48(2), pp.140-144. https://www.stfm.org/FamilyMedicine/Vol48Issue2/Morris140 (Accessed: 30 January 2019).
40 Morrow, G., Rothwell, C., Burford, B. and Illing, J.(2013) ‘Cultural dimensions in the transition of overseas medical graduates to the UK workplace’, *Medical Teacher*, 35(10), pp.e1537-1545. https://doi.org/10.3109/0142159X.2013.802298.
41 Newlands, F., Shrewsbury, D. and Robson, J.(2015) ‘Foundation doctors and dyslexia: a qualitative study of their experiences and coping strategies’, *Postgraduate Medical Journal,* 91(1073), pp.121-126. https://doi.org/10.1136/postgradmedj-2014-132573.
42 Nicholson, S. and Cleland, J.A. (2017) ‘"It’s making contacts": notions of social capital and implications for widening access to medical education’, *Advances in Health Sciences Education Theory and Practice,* 22(2), pp. 477-490. https://doi.org/10.1007/s10459-016-9735-0.
43 Owen L.E., Anderson,S.A. and Dowell, J.S. (2018) ‘Free text adversity statements as part of a contextualised admissions process: a qualitative analysis’, *BMC Medical Education,* 18, 58. https://doi.org/10.1186/s12909-018-1158-6.
44 Patterson, D.G., Baldwin, L.M. and Olsen, PM. (2009) ‘Supports and obstacles in the medical school application process for American Indians and Alaska Natives’, *Journal of Healthcare for the Poor and Underserved*, 20(2), pp.308-29. https://doi.org/10.1353/hpu.0.0150.
45 Razack, S., Maguire, M., Hodges, B. and Steinert, Y.(2012) ‘What might we be saying to potential applicants to medical school? Discourses of excellence, equity, and diversity on the web sites of Canada’s 17 medical schools’, *Academic Medicine*, 87(10), pp.1323-1329. https://doi.org/10.1097/ACM.0b013e318267663a.
46 Razack, S., Lessard, D., Hodges, B.D. and Maguire, M.H *. et al.* (2014) ‘The more it changes; the more it remains the same: a foucauldian analysis of Canadian policy documents relevant to student selection for medical school’, *Advances on Health Sciences Education Theory and Practice*, 19(2), pp.161-181. https://doi.org/10.1007/s10459-013-9468-2.
47 Razack, S., Hodges, B., Steinert, Y. and Maguire, M. (2015) ‘Seeking inclusion in an exclusive process: discourses of medical school student selection’, *Medical Education*, 49(1), pp.36. https://doi.org/10.1111/medu.12547.
48 Rees,C.E. and Monrouxe, L.V. (2011) ‘"A morning since eight of just pure grill": a multischool qualitative study of student abuse’, *Academic Med* *icine,* 86(11), pp.1374-1382. https://doi.org/10.1097/ACM.0b013e3182303c4c.
49 Rothwell, C., Morrow, G., Burford, B. and Illing, J. (2013) ‘Ways in which healthcare organisations can support overseas-qualified doctors in the UK’, *International Journal of Medical Education,* 4, pp.75-82. https://doi.org/10.5116/ijme.515a.2231.
50 Shaw, S.C., Anderson, J.L. and Grant, A.J. (2016) ‘Studying medicine with dyslexia: a collaborative autoethnography’, *The Qualitative Report*, 21(11), pp.2036-2054. https://nsuworks.nova.edu/tqr/vol21/iss11/2. (Accessed: 30 January 2020).
51 Shaw, S.C. and Anderson, J.L. (2017) ‘Doctors with dyslexia: a world of stigma, stonewalling and silence, still?’, *MedEd Publish*, 14;6. https://doi.org/10.15694/mep.2017.000029.
52 Shaw, S.C. and Anderson, J.L. (2018) ‘The experiences of medical students with dyslexia: An interpretive phenomenological study’, *Dyslexia*, 24(3), pp.220-233. https://doi.org/10.1002/dys.1587.
53 Sianou‐Kyrgiou, E. and Tsiplakides, I. (2009) ‘Choice and social class of medical school students in Greece’, *British Journal of Sociology in Education.* 30(6), pp.727-740. https://doi.org/10.1080/01425690903235276.
54 Smith, S., Alexander, A., Dubb, S. and Murphy, K. *et al.* (2013) ‘Opening doors and minds: a path for widening access’ *Clinical Teacher,* 10(2), pp.124-128. https://doi.org/10.1111/j.1743-498X.2012.00616.x.
55 Sockalingam, S., Khan, A.,Tan, A. and Hawa, R. *et al.* (2014). ‘A framework for understanding international medical graduate challenges during transition into fellowship programs’, *Teaching and Learning in Medicine*, 26(4), pp.401-408. https://doi.org/10.1080/10401334.2014.945393.
56 Southgate, E., Kelly, B.J. and Symonds, I.M. (2015) ‘Disadvantage and the ‘capacity to aspire’to medical school’, *Medical Education,* 49(1), pp.73-83. https://doi.org/10.1111/medu.12540.
57 Southgate, E., Brosnan, C., Lempp, H. and Kelly, B. *et al*. ‘Travels in extreme social mobility: how first-in-family students find their way into and through medical education’, *Critical Studies in Education,* 58(2), pp.242-260. https://doi.org/10.1080/17508487.2016.1263223.
58 Spiegel, W., Kamenski, G., Sibitz, I. and Schneider, B. *et al*. (2010) ‘Policy and attitude-related reasons for gender disparity in post allocation for graduate medical education in Austria’, *Medical Teacher*, 32(2), pp.e78-84. https://doi.org/10.3109/01421590903202488.
59 Stergiopoulos ,E., Fernando, O. and Martimianakis, M.A. (2018) ‘"Being on Both Sides": Canadian Medical Students’ Experiences With Disability, the Hidden Curriculum, and Professional Identity Construction’, *Academic Medicine*, 93(10), pp.1550-15 https://doi.org/10.1097/ACM.0000000000002300.
60 Talamantes, E., Gonzalez, K., Mangione, C.M. and Ryan, G. (2016). ‘Strengthening the community college pathway to medical school: A study of Latino students in California’, *Family Medicine*, 48(9), pp.703-710. https://www.stfm.org/FamilyMedicine/Vol48Issue9/Talamantes703 ( Accessed: 30 January 2019).
61 Thackwell, N., Swartz, L., Dlamini, S. and Phahladira, L. *et al*. (2016) ‘Race trouble: experiences of Black medical specialist trainees in South Africa’, *BMC International Health and Human Rights*, 16(1):31. https://doi.org/10.1186/s12914-016-0108-9.
62 Thomas, B., Manusov, E.G., Wang, A. and Livingston, H. (2011) ‘Contributors of black men’s success in admission to and graduation from medical school’, *Academic Medicine.* 86(7), pp.892-900. https://doi.org/10.1097/ACM.0b013e31821d6f3d.
63 Tso, S. (2018) ‘Disabled graduate‐entry medical student experience’, *Clinical Teacher, *15(2), pp.109-113. https://doi.org/10.1111/tct.12653.
64 Van Wyk, J.M., Naidoo, S.S., Moodley, K. and Higgins-Opitz, S.B. (2016) ‘Perceptions of final-year medical students towards the impact of gender on their training and future practice’, *Advances in Medical Education and Practice*, 7, pp.541-550. https://doi.org/10.2147/AMEP.S107304.
65 Varner, K., Mey, L., Mentzel,T and Glazer, G *. et al.* (2018) ‘Pulse Check: Underrepresented Minority Health-Care Student Recruitment and Retention at the University of Cincinnati’, *Journal of College Student Retention*, 20(3), pp.388-405. https://doi.org/10.1177/1521025116675179.
66 Vela, M.B., Kim, K.E., Tang, H. and Chin, M.H. (2010) ‘Improving underrepresented minority medical student recruitment with health disparities curriculum’, *Journal of General Internal Medicine*, 25(2), pp.82-85. https://doi.org/10.1007/s11606-010-1270-8.
67 Wong, A. and Lohfeld, L. (2008) ‘Recertifying as a doctor in Canada: international medical graduates and the journey from entry to adaptation’, *Medical Education,* 42(1), pp. 53-60. https://doi.org/10.1111/j.1365-2923.2007.02903.x.
68 Woolf, K., Cave, J., Greenhalgh, T. and Dacre, J. (2008) ‘Ethnic stereotypes and the underachievement of UK medical students from ethnic minorities: qualitative study’, *BMJ.* 337,a1220. https://doi.org/10.1136/bmj.a1220.
69 Woolf, K., Rich, A.,Viney, R. and Needleman, S. *et al.* (2016) ‘Perceived causes of differential attainment in UK postgraduate medical training: a national qualitative study, *BMJ open.* 6(11):e013429. https://doi.org/10.1136/bmjopen-2016-013429.
70 Wouters, A., Croiset, G., Isik, U. and Kusurkar, R.A.(2017) ‘Motivation of Dutch high school students from various backgrounds for applying to study medicine: a qualitative study. *BMJ open,* 7(5):e014779. https://doi.org/10.1136/bmjopen-2016-014779.
71 Wright, S. (2015) ‘Medical school personal statements: a measure of motivation or proxy for cultural privilege?’, *Advances in Health Sciences Education Theory and Practice*, 20(3), pp.627-643. https://doi.org/10.1007/s10459-014-9550-4.
72 Wyatt,T.R., Egan, S.C. and Phillips, C. (2018) ‘The Resources We Bring: The Cultural Assets of Diverse Medical Students’, *Journal of Medical Humanities*, 39(4), pp.503-514. https://doi.org/10.1007/s10912-018-9527-z.
