# Supplementary material for: The challenge of conducting qualitative research to understand the factors that influence equity in medical education: A scoping review
Source: MedEdPublish (2016). 2020 Apr 30;9:84. [Version 1] doi: 10.15694/mep.2020.000084.1 (PMC10697543; doi:10.15694/mep.2020.000084.1)
Supplement: Peer review history

# Reviewer report: Reviewer response for version 1

**DOI:** 10.21956/mep.20036.r30662

**Published:** 2020-05-22

**Authors:** Hedy S. Wald

**Affiliations:** 1 Warren Alpert Medical School of Brown University

This review has been migrated. The reviewer awarded 4 stars out of 5 It is good to see a scholarly approach to this topic…well referenced…encouraging medical educators to critically reflect on this key topic with a multifactorial approach. This paper reminds us that our research approach in medical education should be nuanced with attention to complexity of factors. It resonates for me with a scoping review by Volpe et al, 2019 in Medical Education that found only 10 of 92 articles considering sociocultural data, ie impact of race, gender, or socio-economic status within consideration of the professional identity formation process. The recommendation of considering strengths of individual learners (a more “appreciative inquiry” approach) within future research is appreciated – we need more of this.

Reviewer Expertise:

NA

No decision status is available

# Reviewer report: Reviewer response for version 1

**DOI:** 10.21956/mep.20036.r30661

**Published:** 2020-05-21

**Authors:** Kieran Walsh

**Affiliations:** 1 BMJ

This review has been migrated. The reviewer awarded 5 stars out of 5 This is a fascinating article on an important subject. There have been concerns about equity in medical education for many years. However these have increased in prominence over the past ten years. A future economic downturn is likely to make the medical education community consider these issues even more deeply. The paper suggests that how we have conducted qualitative research into this subject in the past has had shortcomings and so limits our understanding. One component of equity is the socio-economic background of medical students or those considering a career in medicine. The perceptions of students from poor socio-economic backgrounds is likely to be vastly different to the perceptions of those who run the admissions process. Students from poor backgrounds will look at issues like student debt in a completely different way. We must take account of their perceptions in future research. The authors conclusions could not be more important or more timely.

Reviewer Expertise:

NA

No decision status is available

# Reviewer report: Reviewer response for version 1

**DOI:** 10.21956/mep.20036.r30659

**Published:** 2020-05-14

**Authors:** Ngusha Anyam

**Affiliations:** 1 University of Ibadan

This review has been migrated. The reviewer awarded 4 stars out of 5 A very concise work with persuasive and plausible concepts. Equity, on all levels, is much needed in medical education if medical practice will be inclusive for both individuals and groups. Congratulations to the team!

Reviewer Expertise:

NA

No decision status is available

# Reviewer report: Reviewer response for version 1

**DOI:** 10.21956/mep.20036.r30665

**Published:** 2020-05-08

**Authors:** BALAJI ARUMUGAM

**Affiliations:** 1 TAGORE MEDICAL COLLEGE AND HOSPITAL

This review has been migrated. The reviewer awarded 4 stars out of 5 A well executed qualitative scoping review on understanding the factors that influence the equity in medical education. The review process and the PRISMA flow diagram shows the quality of the review article written. Kudos to the entire team those who involved in this review process. This study has found out that most studies had a focus on only one background characteristic and only two studies had a strengths-based focus on individuals. Recommendations for change was at the ‘policy level’ in ten studies and four studies had learner recommendations for change. No studies with a participatory approach were identified. The recommendations to widen the research for understanding the diversity, focus on all learners at both policy level and local level to enable equity, involving the participatory research methodologies and methods was nicely done by this review article. Once again congrats to the team.

Reviewer Expertise:

NA

No decision status is available

# Reviewer report: Reviewer response for version 1

**DOI:** 10.21956/mep.20036.r30660

**Published:** 2020-05-02

**Authors:** Sarmishtha Ghosh

**Affiliations:** 1 International Medical University

This review has been migrated. The reviewer awarded 4 stars out of 5 This scoping review is a very timely submission as in the Covid-19 era, when equity has become an important issue. The recommendation of the researchers for widening of focus in considering individual learning experiences of learners coming from different backgrounds in terms of schooling and bringing up. Universities need to consider this variation in the background in order to see the students’ successful exit from the medical school. It is also recommended that the students are taken as partners and given a sense of responsibility to patch this unequal state amongst themselves.This paper will give the policymakers in the University and the educators an idea about how to conduct “participatory research in education” include diverse socio-economic groups as research subjects, protecting their confidentiality, thus empowering individuals to make the required amendments in order to enhance equity.The paper is recommended for students, educators, and policymakers.

Reviewer Expertise:

NA

No decision status is available

# Reviewer report: Reviewer response for version 1

**DOI:** 10.21956/mep.20036.r30663

**Published:** 2020-05-01

**Authors:** Nagwa Hegazy

**Affiliations:** 1 Menoufia university

This review has been migrated. The reviewer awarded 4 stars out of 5 This is an informative work. It sheds light on an important area which is equity in medical education using qualitative research. Well illustrated methodology and approach.Important take home messages.

Reviewer Expertise:

NA

No decision status is available

# Reviewer report: Reviewer response for version 1

**DOI:** 10.21956/mep.20036.r30664

**Published:** 2020-04-30

**Authors:** Felix Silwimba

**Affiliations:** 1 University of Lusaka

This review has been migrated. The reviewer awarded 5 stars out of 5 this is a very informative study on equity in medical education. it is equally applicable in low income countries with alot of ethnic groups that fight over control of resources and power. such studies could help in bridging the gap in ethnic diversity in medical practice.

Reviewer Expertise:

NA

No decision status is available
